# Synthesis : Pulling It All Together

**DOI:** 10.1186/1472-6874-4-S1-S30

**Published:** 2004-08-25

**Authors:** Marie DesMeules, Arminée Kazanjian, Heather MacLean, Jennifer Payne, Donna Stewart, Bilkis Vissandjée

**Affiliations:** 1 Centre for Chronic Disease Prevention and Control, Health Canada, 120 Colonnade Rd, Ottawa, Canada; 2 Faculty of Medicine, The University of British Columbia, 5804 Fairview Avenue, Vancouver, Canada; 3 Centre for Research in Women's Health, 790 Bay St., 7th Floor, Toronto, Canada; 4 Centre for Chronic Disease Prevention and Control, Health Canada, 120 Colonnade Rd, Ottawa, Canada; 5 University Health Network, University of Toronto, 657 University Ave, Toronto, Canada; 6 School of Nursing Sciences, University of Montreal, Montreal, Canada

## What the Women's Health Surveillance Report Achieves

This gender-focused Women's Health Surveillance Report is the initial step in developing an effective, sustainable women's health surveillance system in Canada. This report identifies key data gaps in existing national surveys, gaps that must be addressed in order to have an effective women's health surveillance system. It uses data from a variety of national administrative and survey databases to explore sex and gender differences in important areas of women's health. While these data have often been considered "sterile" (they were collected for other purposes and generally lack much of the context needed for gender analysis), the report's authors have used them to provide some insights into disparities in the distribution of determinants of health, health behaviours, health outcomes, and health care utilization for Canadian women, and to identify vulnerable subgroups of women.

This report provides a baseline for monitoring health outcomes, health-related behaviours, and other social and economic issues that affect women's lives. Its focus is a range of health issues that emerged from national consultations with women's health experts.

Many women's health experts, with a variety of perspectives, came together to create the Women's Health Surveillance Report. Expert teams drafted chapters and consulted regularly with the Steering Committee. A broader, external "expert" consultation took place in October 2002. This collaboration will continue through the report's dissemination and evaluation stages, as relevant stakeholders work together to build a comprehensive and effective women's health surveillance system in Canada.

## Some Key Messages: A Crosscutting Perspective

Rather than adopting a crosscutting perspective, the *Women's Health Surveillance Report *presents information on a series of health issues. Below are some key messages from the report with respect to quality of life, the health of Canadian women across the life cycle, and the health of more vulnerable women.

## Quality of Life

The health-related quality-of-life indicators used in this report include self-perceived health, self-reported chronic conditions, and health-adjusted life expectancy (HALE). This report supports the well-known associations between income, education, age-, and self-rated health. As income and education levels increase, women are more likely to rate their health as very good or excellent. Having a partner also seems to contribute to a positive perception of health. On the other hand, older women (65+ years), women not born in Canada, and those who engage in multiple risk behaviours are less likely to report very good or excellent health.

Across all age groups, women are more likely than men to report chronic conditions, comorbidity, and severe and moderate disability. Income, education, smoking status and age are only slightly associated with the prevalence of reported disability. Disabled women, however, are more likely to be single with dependent children, have lower incomes, be unemployed, and have less tangible social support and fewer positive social interactions their male counterparts. Similarly, disabled women over age 45 were less likely to be married than their male counterparts.

Women have lower incomes and less formal education than men, and twice the prevalence of depression. All these conditions are strongly associated with reports of chronic pain. Further, women and men with less social support report pain more frequently.

The chapter on the mortality and life expectancy of Canadian women indicates that women have a longer HALE than men (70.0 years versus 66.7 years). When preventable deaths (e.g. those caused by smoking) are excluded from the analysis, however, this disparity diminishes. As smoking-related deaths and disability continue to increase among women and decrease among men, this sex gap is expected to narrow further.

## Life Cycle

### Young Women

Young women are a subgroup most vulnerable to health risks. Research shows that smoking rates for young women now exceed those of young men – and continue to rise. Young women are some of the largest consumers and abusers of alcohol, with reports of heavy alcohol use among women aged 20–24. Further, adolescent women (15–19 years) are at highest risk of becoming anorexic and/or bulimic, and are more likely to report experiencing violence than women 45+ years. Alarmingly, rates of non-severe violence and emotional abuse are highest in the youngest cohort of women.

Because their cervical cells are still developing and their cervical mucus is more easily penetrated by bacterial organisms that cause disease, adolescent women are at an increased biological risk of contracting chlamydia and gonorrhea. In 2000, the reported incidence of chlamydia and gonorrhea was highest among women aged 15–19 years as compared with older cohorts of women. Further, the greatest increases in positive HIV/AIDS tests attributed to women are found in the youngest age group (15–29 years). The proportion of positive HIV tests attributed to women aged 15–29 has increased steadily, from 14.6% in 1985–1995 to 44.5% in 2001.

Finally, the data suggest that the mental health of young women is a concern. The incidence of depression is higher among young women (18–24 years) than older women, and suicide rates increase over the teen years.

### Women in Midlife

Compared to their younger counterparts, women in midlife (40+ years) begin to be at elevated risk for breast cancer and other gynaecological cancers, such as invasive endometrial cancer and ovarian cancer. Women aged 50 and older face a higher risk of developing cardiovascular disease, the result of both hormonal changes related to menopause and poor health behaviours. Two significant lifestyle changes for women in midlife include decreased physical activity and a greater likelihood of being overweight.

Women in their 40s often begin to experience changes in sexual self-image, sometimes accompanied by diminished sexual desire and decreased sexual responsiveness. These changes are thought to be primarily a result of decreased testosterone levels, although changing social roles for women in midlife may also be a factor.

In general, women aged 45 years and older are less likely to experience depression than their younger counterparts.

### Older Women

As this report shows, as women age, they are at increased risk for breast cancer, cardiovascular disease, gynaecological cancers, osteoporosis, and arthritis. Further, older women are less likely than their younger counterparts to practise positive health behaviours, such as exercising and following a healthy diet. Therefore, it is perhaps not surprising that women aged over 65 years have the highest rates of health care utilization. Notably, age was shown to be a better correlate of primary health care utilization than either sex or geographic location. As age increases, so does the proportion of the population using medication, and the number of medications consumed.

Compared to the amount of data on elderly women's physical health and functioning, little data exists to support an analysis of the non-medical determinants of health, such as social support. This is a substantial data gap, given that elderly women constitute one of the poorest and most vulnerable segments of Canadian society.

Overall, the likelihood of experiencing depression tends to decrease with age. Although psychiatric symptoms, including minor depression and anxiety, increase in the years immediately preceding menopause, these symptoms have been shown to diminish substantially in the post-menopausal years.

## Vulnerable Populations

### Aboriginal Women

Aboriginal women face multiple health burdens, including poor health status, poverty, violence, and substance abuse. There is some agreement that, in Canada, Aboriginal people's health profile resembles that of people in a developing country. Despite this agreement, Aboriginal health – and particularly Aboriginal women's health – remains poorly understood.

The life expectancy of First Nations women is five years below the national average for Canadian women. Belonging to an Aboriginal community is also associated with an increased risk of reporting poor/fair health status. The health practices of First Nations women differ substantially from those of the general female population in Canada. For example, Aboriginal women's smoking rates are double the national average, while alcohol dependence is twice as common among Aboriginal women as it among their non-Aboriginal counterparts. Further, Aboriginal women who are dependent upon alcohol are more likely to experience depression. On the positive side, Aboriginal women are more likely than non-Aboriginal women to be physically active.

Aboriginal women appear to be at greater risk than the general female population for chronic diseases, such as cardiovascular disease, diabetes, arthritis, rheumatism and cervical cancer. Further, they have higher death rates than the general population from ischemic heart disease and stroke. Despite a trend towards an increase in breast cancer among Inuit women (1969–1973 and 1984–1988), it appears that breast cancer rates are lower among Inuit women than the rest of the female Canadian population.

Approximately half of all HIV-positive tests reported among Aboriginal people are from women, compared with 16% for women in the non-Aboriginal population. Further research is required to determine the factors associated with this trend.

Rates of all types of violence, including sexual assault, are much higher among Aboriginal women than non-Aboriginal women. Aboriginal women also have a disproportionate suicide rate: Status Indian teenaged women, for example, are 7.5 times more likely than other Canadian teenagers to commit suicide, and Status Indian women aged 20–29 have a suicide rate 3.6 times that of other Canadian women of similar age. Unfortunately, no national data are available on the prevalence of depression among Aboriginal women.

### Lone Parents

Single mothers are significantly more likely than partnered mothers to be poor and to experience financial stress and food insecurity. These factors may contribute to lone mothers' significantly higher rates of distress (e.g. depressive symptoms), personal stress (e.g. feeling overloaded), and chronic stress. Also of concern is the finding that lone mothers living with young children experience greater rates of severe and non-severe violence and emotional abuse.

### Incarcerated Women

Women in prison have a higher risk of exposure to HIV/AIDS than non-incarcerated women because of injection drug use, needle sharing, and risky sexual behaviour. Other health issues affecting incarcerated women not discussed in this report (e.g. exposure to antibiotic-resistant tuberculosis, hepatitis C, and sexually transmitted infections) require future research.

### Rural Versus Urban Women

This report's findings about the health of rural versus urban women are mixed. On one hand, rural women have significantly higher mortality rates than urban women. For example, mortality rates among rural teenaged girls (15–19 years) are 2.50 times those of their urban counterparts. There are also substantial geographic differences in mortality; for instance, women living in the Northwest Territories have a 60% higher all-cause mortality rate than women living in British Columbia, and a 30% higher mortality rate than that of women in Newfoundland. Rural women are also less likely than women living in urban areas to have had a Pap smear, a clinical breast examination, or a mammogram in the previous six months.

On the other hand, urban women report a higher risk of experiencing physical violence and are more likely than women in rural areas to report being sexually assaulted.

### Ethnic Diversity and Migration

There is a dearth of ethnic-specific national data disaggregated by sex and gender. When ethnicity is available as a variable, it is often presented as broad categories that tend to create homogeneity among diverse sub-groups. The analysis in this report is based on a limited sample of women and men reporting that they were born elsewhere, but currently live in Canada, and/or self-identifying with a specific ethnic group. Our analysis suggests that women who are recent immigrants report better health and are less likely to engage in most health risk behaviours, such as smoking and regular drinking than Canadian born women. As length of residence increases in Canada, women were significantly more likely to report poor health than Canadian-born women. Reported morbidity was also higher for women and men, who had spent more time in Canada, which is similar to the findings of other studies.

## What Else is Needed for a Sustainable Women's Health Surveillance System?

### Other Reports on Women's Health

The United States and several Canadian provinces have prepared women's health reports that provide assessments of women's health status and information that can be used by health decision-makers in developing women's health policy and programs [1-4]. This Women's Health Surveillance Report is unique in that it: (i) uses a Canadian national perspective, and (ii) endeavours to clarify the utility of national secondary data (administrative and survey) in providing gender-relevant information for women's health policy and program decision-making. This important baseline information is necessary for developing a women's health surveillance system.

#### Making the Grade on Women's Health

*A National and State-by-State Report Card*, prepared by the National Women's Law Center in the United States[1], reviewed 32 measures of women's health status and 32 measures of women's health policy. While some of the health issues dealt in the U.S. report with were the same as those in the *Women's Health Surveillance Report*, the U.S. report focused mainly on inequities in access to health care, which is less applicable to the Canadian situation.

Within Canada, different provinces and regions – including Atlantic Canada (1999; updated in 2003), British Columbia (2000), and Ontario (2002) – have reported on women's health [2-4]. Like the *Women's Health Surveillance Report, *these publications provide a gender-relevant perspective on women's health. This approach recognizes that health and its determinants are not distributed equally between men and women or, for that matter, among women themselves, and aims to identify particularly vulnerable groups. While they take different approaches, there are similarities in these reports with respect to issues dealt with and the results obtained.

The Atlantic report showed, through illustrative examples, the utility of a "determinants of health" approach to assessing women's health status in the region[2]. It addressed issues including socio-economic determinants of health, lifestyle and preventive factors, and disease. The report noted the highly interactive nature of the determinants of health and identified some significant data gaps and limitations. The British Columbia report used the six provincial health goals and their corresponding objectives and indicators as a framework for the report[3]. Like the Atlantic report, it was intended to demonstrate an alternative approach to women's reporting. The Ontario report provided a comprehensive description of the current state of women's health in the province. It looked at health and the determinants of health, and was organized around five themes: demographics, morbidity indicators, reproductive health, health behaviours, and subpopulations[4].

These reports, and the research teams that worked on them, provide opportunities for further insight into and collaboration towards strengthening women's health surveillance in Canada.

### Beyond this Report

During the consultation process and subsequent development of the *Women's Health Surveillance Report, *several stakeholders and women's health experts suggested more or different women's health issues that could/should have been included. Not all these topics are discussed here, for a variety of reasons. For example, there are data available on respiratory diseases among women, which should be addressed in future reports. In some cases, such as abortion and hysterectomies, insufficient data are available for a comprehensive analysis. Also there are other important issues such as anxiety in women and pelvic pain for which there are no appropriate data. There is clearly a need to continue the process of consensus building, and further the development and validation of women's health indicators. The agreement on a framework and a core set of gender-sensitive women's health indicators will be an important step towards establishing a comprehensive and valid scope for future reports.

Such a framework and indicators would facilitate appropriate and useful data collection. While it has been possible to provide some sex- and gender-relevant data analysis for all issues covered in this report, almost all chapter authors have commented on the limitations of the secondary data available for constructing the most appropriate gender variables. The authors of several chapters expressed the need for more suitable data for gender-sensitive analysis, particularly the following:

• More contextual data pertaining to women's circumstances, such as women's roles (e.g. employee, wife, mother, caregiver) and women's use of health care resources (qualitative and quantitative research should complement each other and help to determine the most useful contextual variables).

• Longitudinal data that would allow a better understanding of the links between health behaviours and health outcomes.

• Representative samples in health surveillance systems to reflect the diversity of the Canadian population with respect to ethnic background and length of residence in Canada, especially to assess the needs of recent immigrants.

• Longitudinal data that would provide information of the influence on changing socio-economic environments, of transition experiences and their interaction with gender roles, ethnicity, migration experiences and health.

• More contextual Aboriginal data.

Consideration was given to standardizing the broad social concepts (e.g. age, education, employment, housing, ethnicity, immigrant groups, etc.) used to examine gender in this report. Given the diversity of the issues addressed, however, it seemed inappropriate to categorize these variables arbitrarily. It was decided, instead, to use the results of the report as a basis for recommending the most appropriate categories for gender-based analysis. Accordingly, a "concept dictionary" could be developed, providing proposed categorizations for their use in future gender-relevant analyses.

The analyses, and this report, have focused primarily on the individual. The importance of more "upstream" political, social, cultural, and economic determinants of health, however, must be acknowledged. As well, indicators that measure, monitor, and report on these determinants – and on risk factors, exposures, interventions, and health outcomes – must be built in to any sustainable women's health surveillance system.

Although a systematic life-course approach was not taken, authors of various chapters have chosen to focus on women in specific age groups. A systematic investigation of women's health risks across the different stages of life (early life, childhood, adolescence, and early, middle, and late adult life), however, can provide insights into the biological, psychosocial, and social factors that interact to influence women's health. Our understanding of women's health would benefit from increased knowledge in this area, and more emphasis should be placed on health across life stages in future reports.

A sustainable women's health surveillance policy development cycle, as envisioned by the Advisory Committee on Women's Health Surveillance[5], requires interdisciplinary input (from researchers, health practitioners, data collectors, analysts and interpreters, policy and program developers, and communicators) at all stages. Input from policy and other decision-makers can help guide data collection and analysis. Similarly, an understanding of the needs of policy and communications teams could help focus both data analysis and the reporting of results. As well, trends and important health patterns identified by surveillance experts can help inform and focus policy and program decision-making. The more inclusive the surveillance system, and the more multifaceted the perspectives and approaches to it, the more likely it is to be sustainable and relevant to policy.

### Next Steps

The core research team identified the following next steps in the process of developing a sustainable, national women's health surveillance system in Canada:

• Evaluating this report to determine the extent to which it is a practical and useful tool for women's health policy and program development

• Compiling the longer-term recommendations from stakeholders and experts into a format that can inform future women's health reports. Here, it would be useful to include a systematic assessment of the recommendations' possible effectiveness.

• Further work with diverse partners to develop and validate a gender-sensitive framework for and indicators of women's health that can be used in the preparation of future reports. These tools would facilitate the standardization of data and the comparison of data between the groups that use them. Further, they would assist in setting priorities.

• Continued engagement of women's health experts and other stakeholders in the process of refining a gender-sensitive model of surveillance that can form the evidence base for women's health policy and programming.

The *Women's Health Surveillance Report *aims to provide a useful tool for examining women's health, a tool that can help policymakers set specific health goals for Canadian women, improve Canadian women's health, and inform the development of Canada's national health goals.
